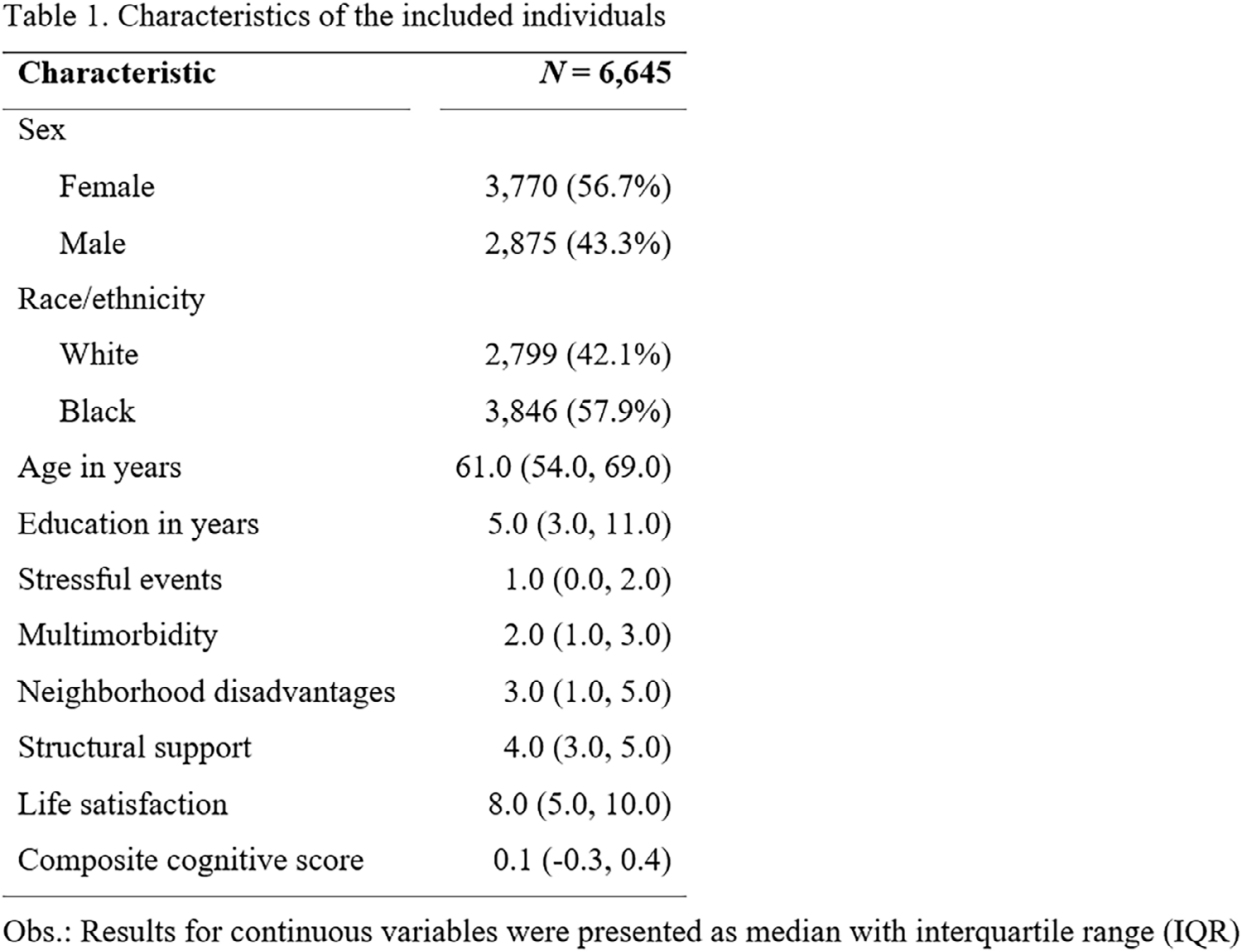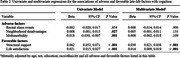# The buffering effect of structural support and life satisfaction on cognition is independent of adverse events at older ages: Findings from the ELSI‐Brazil study

**DOI:** 10.1002/alz70860_101920

**Published:** 2025-12-23

**Authors:** Ari Alex Ramos, Liana Machado, Carolina Godoy, Lucas Martins Teixeir, Maria Fernanda Lima‐Costa, Marcia Regina Cominetti, Cleusa P Ferri

**Affiliations:** ^1^ Federal University of São Carlos, São Carlos, São Paulo, Brazil; ^2^ Universidade Federal de São Paulo (UNIFESP), São Paulo, São Paulo, Brazil; ^3^ Universidade Federal de São Carlos, São Carlos, São Paulo, Brazil; ^4^ Universidade Federal de São Paulo, São Paulo, São Paulo, Brazil; ^5^ Universidade Federal de São Paulo (UNIFESP), São Paulo, São Paulo/SP, Brazil; ^6^ Fiocruz, Belo Horizonte, MG, Brazil; ^7^ Global Brain Health Institute, Dublin, Dublin, Ireland

## Abstract

**Background:**

Emerging literature suggests adverse later‐life events are associated with an increased risk of cognitive impairment and dementia. Conversely, richer structural support (here defined as social networks and contacts) and higher levels of life satisfaction have shown a buffering effect against neurocognitive disorders in older adults. Herein, we sought to cross‐sectionally examine the associations of later‐life adverse (recent stressful events, neighborhood disadvantages, and multimorbidity [number of chronic diseases]) and favorable (structural support and life satisfaction) factors with cognitive outcomes.

**Method:**

This population‐based study enrolled a nationally representative sample of 6,645 older adults aged 50 and over with completed data, drawn from the Brazilian Longitudinal Study of Aging (ELSI‐Brazil). Linear regression models characterized the association of each relevant factor with the primary measurable outcome (composite cognitive scores). Moreover, we analyzed whether sex and race/ethnicity serve as effect modifiers in these associations.

**Result:**

Increased structural support (*β* = 0.030, 95% CI [0.021, 0.038], *p* < .001) and higher levels of life satisfaction (*β* = 0.014, 95% CI [0.009, 0.019], *p* < .001) were associated with better cognitive performance, independent of age, sex, education, race/ethnicity, and all adverse factors combined. Notably, a statistically significant structural‐support‐by‐race/ethnicity interaction (*β* = 0.021, 95% CI [0.002, 0.039], *p* = .029) revealed that the independent protective effect of structural support was twice as high in Black individuals (*β* = 0.039, 95% CI [0.026, 0.052], *p* < .001) compared to White participants (*β* = 0.019, 95% CI [0.006, 0.031], *p* = .003). No significant sex‐based interaction was found.

**Conclusion:**

Findings suggest structural support and life satisfaction offer robust positive effects on cognitive health in older adults, independent of later‐life adversities, with structural support showing stronger benefits among Black individuals. Public health initiatives should prioritize building community‐based support structures, while addressing factors that contribute to life satisfaction in later adulthood.